# Exploring views on what is important for patient-centred care in end-stage renal disease using Q methodology

**DOI:** 10.1186/s12882-015-0071-z

**Published:** 2015-05-28

**Authors:** Jane M. Cramm, Laszlo Leensvaart, Mathilde Berghout, Job van Exel

**Affiliations:** Institute of Health Policy and Management, Erasmus University Rotterdam, Rotterdam, The Netherlands

## Abstract

**Background:**

This study aimed to explore views on what is considered important for Patient-Centred Care (PCC) among patients and the healthcare professionals treating them in a haemodialysis department.

**Methods:**

Interviews were conducted among 14 patients with end-stage renal disease receiving dialysis and 12 healthcare professionals (i.e. 2 doctors, 4 staff members, and 6 nurses) working at a haemodialysis department. Participants were asked to rank-order 35 statements representing eight dimensions of PCC previously discussed in the literature. Views on PCC, and communalities and differences between them, were explored using by-person factor analysis.

**Results:**

Four views on what is important for PCC in end-stage renal disease were identified. In viewpoint 1, listening to patients and taking account of their preferences in treatment decisions is considered central to PCC. In viewpoint 2, providing comprehensible information and education to patients so that they can take charge of their own care is considered important. In viewpoint 3, several aspects related to the atmosphere at the department were put forward as important for PCC. In viewpoint 4, having a professional or acquaintance that acts as care coordinator, making treatment decisions with or for them, was considered particularly beneficial. All views agreed about the relative importance of certain PCC dimensions; the patient preferences and information and education dimensions were generally considered most important, while the family and friends and the access to care dimensions were considered least important.

**Conclusions:**

The four views on PCC among patients in a haemodialysis department and the professionals treating them suggest that there is no one size fits all strategy for providing PCC to patients with end-stage renal disease. Some patients may benefit from educational interventions to improve their self-management skills and place them in charge of their own care, whereas other patients may benefit more from the availability of a care coordinator to make decisions for them, or with them. Furthermore, our results suggest that not all eight dimensions of PCC need to be given equal consideration in the care for patients with end-stage renal disease in order to improve patient outcomes.

## Background

Since the Institute of Medicine identified Patient-Centred Care (PCC) as one of six domains of quality of care in 2001, PCC has received much more attention in research and clinical practice [1]. Perhaps the most comprehensive study about what constitutes PCC was conducted by the Picker Institute in conjunction with the Harvard School of Medicine [2,3]. In this study, eight dimensions of PCC were distinguished: ‘respect for patients’ values’, ‘preferences and expressed needs’, ‘provision of information and education’, ‘access to care’, ‘emotional support to relieve fear and anxiety’, ‘involvement of family and friends’, ‘continuity and secure transition between healthcare settings’, ‘physical comfort’ and ‘coordination of care’ [2-9].

There is considerable evidence for the benefits of investing in the improvement of these eight PCC dimensions for quality of care. In a systematic review, Rathert and colleagues [4] concluded that organisations with higher scores on multiple dimensions of PCC also reported better patient as well as organizational outcomes. However, little research has been conducted on the relative importance of the different dimensions of PCC for quality of care, and therefore it currently remains unclear whether each dimension delivers a similar contribution to quality of care. Furthermore, the relative importance of these dimensions may differ among patient groups and care settings; for instance, patients with end-stage renal disease who undergo intensive dialysis treatment in hospital are known to have various levels of self-care [10], which may result in different physical as well as mental health-care needs and preferences. A broader understanding of what is considered important for PCC for patients with end-stage renal disease receiving dialysis could help to improve the organisation and provision of care, which may be expected to lead to better quality of care and patient outcomes. However, the effectiveness of different care delivery strategies may differ among patients, depending on their needs and preferences. Therefore, this study was conducted to explore views on what is considered important for PCC among patients with end-stage renal disease and professionals in a haemodialysis department.

## Methods

### Q methodology

Views on what is important for PCC were examined using Q methodology, which combines aspects of qualitative and quantitative research methods to study subjectivity [11]. Q methodology has been used a number of times before to study patients’ views on their disease [e.g. 12,13] or treatment [e.g. 14-16].

Since this study did not include an intervention of any kind but rather investigated views on PCC the study does not fall under the scope of the Medical Research Involving Human Subjects Act (WMO) and therefore did not need to undergo a review by an accredited medical research ethics committee (MREC) or the Central Committee on Research Involving Human Subjects (CCMO). Informed consent was sought by the interviewer and granted by the respondents by taking part in the research for quality improvement. Results of the research will be used by the haemodialysis department to further improve quality of care and patient centeredness. Participants’ anonymity and confidentiality were ensured.

#### Development of the research instrument

The eight dimensions of PCC were used as a reference framework for developing the research instrument. A review of additional literature on PCC [e.g. 5-9] revealed no additional dimensions. The eight PCC dimensions used in this study are described in more detail in Table 1. The four authors individually developed statements representing the eight dimensions of PCC. All authors reviewed the developed materials and discussed them in a number of consecutive group meetings. Agreement was reached on a final set of 35 statements (Table 2).

**Table 1:** The eight dimensions of PCC

Dimension	Description
1) Respect for patients’ values, preferences, and expressed needs	Patients have indicated that they feel the need to be treated with dignity and respect and to be seen as whole persons, not merely as a disease or functional impairment [12,13]. Whole-person care is a concept requiring professionals’ understanding of each patient as a whole by taking the time to really get to know the patient and his/her values and preferences, thereby improving the patient’s quality of life [15,16,18,19]. To enhance PCC, healthcare professionals should involve patients in decisions about their care and support them in setting and achieving their own treatment goals [12,13].
2) Information and education provision	Patients expressed the fear that information would be withheld from them [12,13]. The provision of complete information to patients about all aspects of their care is thus necessary. Patients should have access to their care records and be in charge of their care. Open communication between patients and healthcare professionals, which requires professionals to possess high-quality communication skills, is also necessary [18,19].
3) Access to care	Access to care involves patients’ ability to make appointments promptly and easily, the availability of healthcare professionals, support and navigation for illiterate patients, and consideration of cultural differences [18]. Hospitals must be accessible to all patients, (including those with mobility issues), post clear directions in several languages, and have a clear, user-friendly scheduling system in place [12,13].
4) Emotional support to relieve fear and anxiety	Patients sometimes experience anxiety about the impact of their illness on their lives and those of their loved ones. PCC requires professionals to pay attention to this type of anxiety [12,13].
5) Involvement of family and friends	Depending on the seriousness of the condition, an illness can affect not only the patient, but also his/her family and friends. One example is the lengthy hospitalisation of a child. In such cases, PCC may be improved by the availability of accommodations for relatives near the hospital, the involvement of relatives in decisions about the patient’s care, and attention to the role and needs of informal caregivers [12,13].
6) Continuity and secure transition between healthcare settings	Especially in the hospital setting, continuity and secure transition between healthcare settings have been identified as important aspects of PCC [12,13]. These concerns refer to in-hospital transfers (e.g. from the intensive care unit to other departments), but also to transitions to rehabilitation centres, nursing homes, and long-term care facilities. Smooth transitions require the transfer of all relevant patient information; ensuring that patients are well informed about where they are going, what care they will receive, and who their contact person will be; and the provision of skilled advice about care and support at home after hospital discharge.
7) Physical comfort	Patients’ physical comfort should be supported effectively. Care areas should be clean and comfortable, patients' privacy must be respected, pain should be effectively managed, and healthcare professionals should take patients' preferences about support and their daily living needs into account [12,13,16].
8) Coordination of care	Patient care should be well coordinated among professionals (teamwork in care delivery). Healthcare professionals should be well informed so that patients need to tell their stories only once; patients should have a primary contact person who knows everything about their condition and treatment [12,13,18,19].

**Table 2:** Q set statements

Dimension	Examples	Statements
Patients’ preferences	- Providing care in a respectful atmosphere with dignity and respect	1. Healthcare professionals treat patients with dignity and respect.
	- Focus on quality of life issues / whole-person care	2. Healthcare is focused on improving patients’ quality of life.
		3. Healthcare professionals take patients’ preferences into account.
	- Informed and shared decision making / patient participation and involvement	4. Healthcare professionals involve patients in decisions about their care.
	- Personal goals and outcomes	5. Patients are supported in setting and achieving their own treatment goals.
Physical comfort	- Pain management	6. Healthcare professionals pay attention to pain management.
	- Assistance with daily living needs	7. Healthcare professionals take patients’ preferences for support and daily living needs into account.
	- Hospital surroundings and environment	8. Patient areas in hospital are clean and comfortable.
		9. Patients have privacy in the hospital.
Coordination of care	- Coordination and integration of care	10. Healthcare professionals are well informed; patients need to tell their story only once.
		11. Patient care is well coordinated among professionals.
	- Spokesperson for navigation through the system	12. Patients know who is coordinating their care.
		13. Patients have a primary contact who knows everything about their condition and treatment.
	- Teamwork	14. Healthcare professionals work as a team in care delivery to patients.
Emotional support	- Anxiety about consequences of the changed situation	15. Healthcare professionals pay attention to patients’ anxiety about their situations.
	- Creating support systems	16. Healthcare professionals involve relatives in emotional support of the patient.
	- Anxiety about the impact of one’s illness on one’s family and loved ones	17. Healthcare professionals pay attention to patients’ anxiety about the impact of their illness on their loved ones.
Access to care	- Access to location / specialist	18. The hospital is accessible for all patients.
	- Availability of transportation	19. Clear directions are provided to and inside the hospital.
	- Clear instructions provided on how and when to get referral	
	- Ease of scheduling appointments	20. Appointment scheduling is easy.
	- Waiting time	21. Waiting times for appointments are acceptable.
	- Language barrier	22. Language is not a barrier for access to care.
	- Cultural differences	
Continuity and transition	- Understandable, detailed information regarding all aspects of care	23. When a patient is transferred to another ward, relevant patient information is also transferred.
	- Coordination and planning of ongoing treatment	24. Patients who are transferred are well informed about where they are going, what care they will receive, and who their contact person will be.
	- Provide information regarding access to support after hospital discharge	25. Patients receive skilled advice about care and support at home after hospital discharge.
Information and education	- Information on all aspects of care (e.g. clinical status, progress, prognosis, care processes)	26. Patients are well informed about all aspects of their care.
	- Information on processes of care	27. Patients can access their care records.
	- Information and education to facilitate autonomy and self-care	28. Patients are in charge of their own care.
		29. Healthcare professionals support patients to be in charge of their care.
	- Open communication between patient and caregiver	30. Open communication between patients and healthcare professionals occurs.
	- Skills and knowledge of caregiver	31. Healthcare professionals have good communication skills.
Family and friends	- Accommodation	32. Accommodations for relatives are provided in or near the hospital.
	- Respect for role in decision making	33. Healthcare professionals involve relatives in decisions about the patient’s care.
	- Support for family as caregivers	34. Healthcare professionals pay attention to loved ones in their role as the patient’s caregivers.
	- Recognition of the needs of family and friends	35. Healthcare professionals pay attention to the needs of the patient’s family and friends.

**Table 3:** Rank scores of statements for views on patient-centred care

Domain	Statement		View on PCC			
			1	2	3	4
Patient’s preferences	1	Healthcare professionals treat patients with dignity and respect.	+4	+3	+2	+4
	2	Healthcare is focused on improving the quality of life of patients.	+3	+3	0	+1
	3	Healthcare professionals take into account patient preferences.	0	+1	+2	0
	4	Healthcare professionals involve patients in decisions regarding their care.	+4	+3	+3	−1**
	5	Patients are supported to set and achieve their own treatment goals.	−1**	+4**	+1**	−4**
Physical comfort	6	Healthcare professionals pay attention to pain management.	+2	+2	−2**	0
	7	Healthcare professionals take patient preferences for support with their daily living needs into account.	−2	+1**	−1	−2
	8	Patient areas in hospital are clean and comfortable.	−2	−1	+4	+3
	9	Patients in hospital have privacy.	+1	−1	−1	0
Coordination of care	10	Healthcare professionals are well-informed; patients need to tell their story only once.	0*	−1*	+2*	−3*
	11	Patient care is well-coordinated between professionals.	+1	0	0	−1
	12	Patients know who is coordinating their care.	−1	0**	−2	−3
	13	Patients have a first point of contact who knows everything about their condition and treatment.	+2**	0	−1	+4**
	14	Healthcare professionals work as a team in care delivery to patients.	+1	0	+3**	+2
Emotional support	15	Healthcare professionals pay attention to patients' anxiety about their situation.	+1	+1	−1**	+2
	16	Healthcare professionals involve relatives in the emotional support of the patient.	−4	−2	0	0
	17	Healthcare professionals pay attention to patients' anxiety over the impact of their illness on their loved ones.	−2	0	+1	−1
Access to care	18	The hospital is accessible for all patients.	+2*	−3**	0	0
	19	Clear directions are provided to and inside the hospital.	−3	−3	−4	−1
	20	It is easy to schedule an appointment.	0**	−4	−2	−3
	21	Waiting times for an appointment are acceptable.	0**	−4*	−3	−2
	22	Language is not a barrier for access to care.	0	−2*	0	−1
Continuity and transition	23	When a patient is transferred to another ward, relevant patient information is transferred as well.	+2	+1	−1	−2
	24	Patients who are transferred are well-informed about where they are going, what care they will receive and who will be their contact person.	0	−1	0	0
	25	Patients get skilled advice about care and support at home after hospital discharge.	0	+1	0	+2*
Information and education	26	Patients are well-informed about all aspects of their care.	+3	+2	+1	+3
	27	Patients can access their care records.	−4	0**	−2	−4
	28	Patients are in charge of their own care.	−1**	+4	+4	1**
	29	Healthcare professionals support patients to be in charge of their care.	−1*	+2	+1	0
	30	There is open communication between patient and healthcare professionals.	+3	0**	+3	+3
	31	Healthcare professionals have good communication skills.	+1	+2	+2	+1
Family and friends	32	Accommodation for relatives is provided in or nearby the hospital.	−3	−3	−4**	+1**
	33	Healthcare professionals involve relatives in decisions regarding the patient's care.	−2	−2	+1	+2
	34	Healthcare professionals pay attention to loved ones in their role as carer for the patient.	−1	−1	−3*	+1**
	35	Healthcare professionals pay attention to the needs of family and friends of the patient.	−3	−2	−3	−2

*Distinguishing at P < 0.05, **distinguishing at P < 0.01

#### Data collection

This study was performed in the haemodialysis department of the Maasstad Hospital in Rotterdam, the Netherlands [17]. This is one of the largest in the Netherlands, with 25,274 haemodialysis treatment sessions performed annually (2013). To ensure wide representation of views on PCC, we used purposive sampling to recruit patients and professionals with different background characteristics (e.g. patient age, educational level; professional occupational background).

**Fig. 1:**
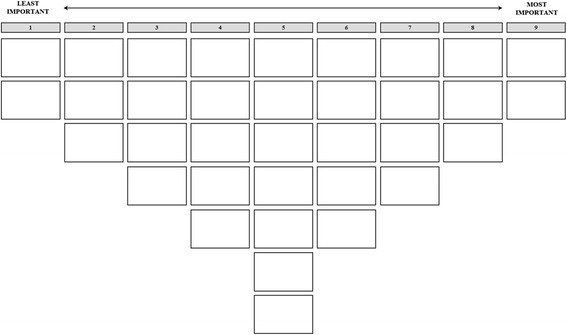
Score sheet used for ranking the statements

Interviews with patients and professionals (45 ~ 60 min each) were all conducted in the hospital by one of the authors (LL). Patients were interviewed during their dialysis at the department. This means they were sitting in a chair, connected to the machine. To make sure the patients could fill in the score sheet themselves, it was presented at a movable cart. Professionals on the other hand were individually invited in a separate meeting room at the department. This was done during their shift or for some employees during a break, especially provided by the team manager to participate in the study. All interviews were recorded and transcribed. Respondents were asked to rank the 35 statements (Table 2) according to perceived importance for PCC. First, respondents were asked to read all the statements and sort the cards on which these were printed into piles representing aspects they considered important, neutral, or important for PCC. Respondents were then asked to read the statements in a pile once again and to rank them using a score sheet ranging from 1 (least important) to 9 (most important; see Fig. 1). Respondents consecutively ranked the statements in the agree pile (on the right side of the score sheet), the disagree pile (on the left side) and, finally, the neutral pile (on the remaining spots in the middle area). After placing all the statements on the score sheet, respondents were asked to elaborate on their ranking, especially on the aspects of PCC that they identified to be most and least important for PCC. To check the comprehensiveness of the statement set, respondents were asked whether any important aspect of care was missing. Finally, background information (e.g. age, marital status, educational level) was gathered.

#### Analysis of the data

The quantitative part of the analyses consisted of by-person factor analysis using common techniques in Q methodology (i.e., centroid factor extraction, varimax factor rotation) was performed to identify groups of respondents who had ranked the statements in a similar way. For each of the identified factors a weighted average ranking of the statements was computed based on the rankings of statements by respondents associated with the factor, weighted by their correlation coefficient with the factor. Statements that received a statistically significantly different ranking in a factor as compared to all the other factors were identified as distinguishing statements for that factor. Statements that did not distinguish statistically significantly between any pair of factors were identified as consensus statements. The weighted average rankings of statements for each factor were interpreted and described as distinct views on PCC. Finally, the qualitative part of the analyses consisted of the explanations participants gave during the follow-up interview. These explanations were used to interpreted and verify the different views found in the quantitative part of the analyses. At first the transcribed interview texts were read through a couple of times to understand the overall meaning. Secondly, the interviews were read separately for each of the four views to interpreted and verify the different views found. Some literal quotes from respondents were used as examples in the description of the views. Analyses were conducted using PQ Method 2.11 software [18].

## Results

Interviews were held with 26 participants: 14 patients with end-stage renal disease on dialysis, 6 nurses, 4 staff members (i.e. 1 team leader, 1 policy advisor, 1 quality advisor, 1 social worker), and 2 nephrologists. The mean age of respondents was 58 (range, 23–84) years. Twelve respondents were female and 14 were male; education levels ranged from primary school to university.

Factor analysis revealed four distinct views on PCC. Together, they explained 47 % of the variance in the correlation matrix. Factor arrays are shown in Table 3. Viewpoints 1 and 2 were defined by professionals and patients, whereas viewpoints 3 and 4 were defined exclusively by patients.

### Viewpoint 1

Respondents with this perspective believed that consideration of *patients’ preferences* was an important aspect of PCC, as evidenced by the importance of treating patients with dignity and respect (item 1; staff member 4 and patient 11 stated that professionals should ‘take the patient seriously and respect their choices’), improving quality of life (item 2), and involving patients in decisions about their care (item 4).

Respondents with this viewpoint also considered the provision of *information and education* to patients (items 26 and 30) to be important for PCC; these aspects required open communication (nurse 5: ‘Good communication is needed to gain trust and reduce the number of mistakes’) and information provision during personal face-to-face visits, not merely through patients’ access to their records. Respondents with this viewpoint felt that patients being in charge of their own care (item 28) and receiving support to achieve this goal (item 29) were less important. Thus, this viewpoint appears to place more importance on patients being seen and listened to, rather than being in charge of their care themselves.

People with this viewpoint scored significantly higher on three aspects of *access to care* (items 18, 20, and 21). They also found hospital accessibility and the ease of appointment making more important than did those with other viewpoints.

### Viewpoint 2

Respondents with this viewpoint linked *patients’ preferences* closely to PCC. Most striking was the importance of supporting patients in setting and achieving their own treatment goals (item 5), as demonstrated by the following statement: ‘Patients need to be informed, so they can make the right decision about what is going to happen next in their care delivery’ (nurse 4). Treating patients with dignity and respect (item 1), quality of life (item 2), and patients’ involvement in decision making (item 4) were also considered to be important aspects of PCC.

Respondents with this viewpoint also identified the provision of *information and education* as an important aspect of PCC. They especially felt that patients being in charge of their own care (item 28) was very important, as demonstrated by the following statements: ‘The patient has to make the final decision’ (nurse 1); ‘The patient should be autonomous’ (staff member 2); ‘Everything I can decide, I will decide’ (patient 13); and ‘People do not have to pay attention to the things that I can still do myself’ (patient 8). The importance of patients’ autonomy and control among respondents with this viewpoint was also evidenced by high ranking of items 12 (patients know who is coordinating their care) and 27 (patients can access their care records).

*Access to care* was least important for PCC among respondents with viewpoint 2. They considered hospital accessibility (item 18), clear directions (item 19), ease of appointment scheduling (item 20), waiting times (items 21), and the overcoming of a language barrier (item 22) to be relatively unimportant.

### Viewpoint 3

Respondents with this viewpoint considered the *atmosphere at the department* where they receive treatment to be the most important aspects of PCC. Most important for a good atmosphere mentioned by the respondents are an open, friendly, intimate and trustworthy mood during a patients stay. Items ranked as most important related to the context of care provision and were from several dimensions of PCC: patient’s preferences (item 4), physical comfort (item 8), coordination of care (item 14), and information and education (items 28 and 30). The importance of context is exemplified by the following statements: ‘The atmosphere at the department is of great importance to me’ (patient 4); ‘It is important that the department is clean’ (patient 5); and ‘Professionals working as a team increase the right mood/atmosphere at the department, which also decreases the chance of making mistakes’ (Patient 10)*.* Patients with this viewpoint also wanted to be well informed so that they could make decisions themselves: ‘you have to be able to make your own decisions in care delivery' (patients 1)’ and ‘I want to be informed about everything’ (patient 5)*.*

Respondents with this viewpoint considered aspects related to *family and friends* (items 32, 34, and 35) and some aspects of access to care (items 19 and 21) to be least important. The low importance of attention to relatives’ needs (item 35) and patients’ anxiety (item 34) can be explained by the following statement: ‘Patients should be the main focus of care delivery, just a little attention may in some cases be needed for close relatives’ (patient 4). From the background data we observed that respondents defining this view lived near the hospital, which may explain the low priority they placed on issues related to access of the hospital and accommodation for relatives.

### Viewpoint 4

The most important aspects of PCC for respondents with this viewpoint were spread across the dimensions *patient’s preferences* (item 1), *physical comfort* (item 8), *coordination of care* (item 13), and *information and education* (items 26 and 30). Respondents with this view want to be treated with respect and dignity and have a primary contact person for their care: ‘It is nice to have one person I can tell everything to, and thereby build a relationship of confidence’ (patient 2)*.* In addition, being well informed and having open communication was considered important: ‘Everything has to be clearly explained’ (patient 9) and ‘I want to be informed well in a way I understand’ (patient 14)*.* Explanations like these suggest that patients need *guidance* in their care delivery. This interpretation is supported by the aspects that they found least important: items 5 (patient’s preferences), 10 and 12 (coordination of care), 20 (access to care), and 27 (information and education). In this view, having to tell your stories more than once is not an issue, because having contact with professionals is appreciated. In addition, setting own treatment goals, having access to medical records, or knowing who is coordinating care is relatively unimportant, as long as it happens: ‘It is not important that I decide myself, in the end the doctor always knows best’ (patient 9)*.*

Contrary to the other viewpoints, respondents with viewpoint 4 considered items related to the *family and friends* dimension to be important for PCC. More than others, they appear to need a trusted healthcare professional or a close relative or friend to talk to about issues related to their care, and make treatment decisions with or for them. From the interviews and background data we observed that respondents defining this viewpoint generally were more dependent on others when it comes to making decisions in their care delivery and they were more often lower educated.

### Consensus between views

Five of the 35 statements from four dimensions of PCC were ranked similarly in all four views. First of all, being well informed about their care (item 26) and professionals having good communication skills (item 31) were considered as fairly important in all views, although perhaps for different reasons. In view 1, exchange of information is considered important so that professionals can make decisions taking account of the needs and preferences of patients, while in view 2 it is important because it enables patients to take charge of their own care. In view 3, communication is an important part of creating a trusting and comfortable atmosphere at the department. In view 4, the need for information and communication appears to come from a sense of insecurity and need for guidance in treatment decisions.

In all viewpoints, item 3 (healthcare professionals take into account patient preferences) was considered as not really important, unlike all other items from the patient’s preferences dimension of PCC. From the interviews we learned that most respondents acknowledged that professionals’ ability to take patients’ preferences into account was limited and regarded it as a luxury. Item 24 (patients who are transferred are well informed about where they are going, what care they will receive, and who their contact person will be), from the continuity and transitions domain of PCC received a neutral score in all views, possibly due to the infrequency with which patients with end-stage renal disease are transferred from the haemodialysis department. Finally, there was consensus on the unimportance of the needs of family and friends (item 35) for PCC. All items of the family and friends dimension of PCC received low scores in views 1, 2 and 3 support of family and friends was considered a secondary goal of care delivery, which they believed should focus on the patient. However, in view 4 these items were considered significantly more important (except item 35), as in this view patients depend much more on the support and involvement of their loved ones.

## Discussion

In this study four views on what is important for PCC were identified, suggesting that different patients may benefit from a different type of care. Patients who adhere to viewpoint 1 are likely to benefit from empathic healthcare professionals. Previous research has shown that the use of reflective statements increases patient satisfaction [19]. Respondents with viewpoint 2, in contrast, considered communication (i.e. understandable provision of relevant information and education) to be more important for PCC than empathy. A setting and delivery method that fit a patient’s learning style (e.g. auditory, visual, or kinaesthetic) may be expected to enhance PCC [20]. Patients with viewpoint 3 also considered communication to be an important aspect of PCC, but additionally identified the importance of a supportive context of care delivery. In support of this finding, Adekanye and colleagues [21] for example found significant positive relationships between patient satisfaction and staff promptness, staff communication level, environmental cleanliness, and comfortable facilities. The improvement of PCC for patients with viewpoint 3 thus requires not only high-quality care delivery, but also hospitals’ attention to the context in which care is delivered. Patients holding viewpoint 4, which appears more common among those who are more dependent concerning decision making and less-educated, may benefit considerably from having a care coordinator to make decisions for them.

The results of this study indicate that the enhancement of PCC in hospital departments and thereby the improvement of patient outcomes does not always require investment in all eight PCC dimensions. Patients with end-stage renal disease, for example, all agreed on the importance of the patient’s preferences and the information and education dimensions, and the relative unimportance of the family and friends and the access to care dimensions. Investment in the patient’s preferences and the information and education dimensions is therefore expected to enhance PCC for patients with end-stage renal disease. Previous studies have acknowledged the importance of these dimensions [22-24]. A model of bedside reporting, with shift-to-shift reports provided at the patients’ bedsides, was found to increase patients’ involvement and ensure that they received better information about their health status, medical plans, and treatment progress [25]. Patients with end-stage renal disease may thus benefit from the implementation of such a model. They may also benefit from healthcare professionals’ behaviours, such as immediacy and perceived listening, which have also been found to be positively associated with patient satisfaction [26].

Patients and professionals participating in the present study perceived no added value of patients’ access to medical records. In contrast, Davis Giardina et al. [27] reported that such access enhanced patients’ perception of control. Although this item was slightly more important in view 2 than in the other views, it was regarded fairly unimportant overall. Respondents felt that the information contained in medical records was too complex to understand and would only raise questions, leading to unnecessary confusion. Thus, access to medical records does not appear to be an important focus for efforts to enhance PCC, unless the comprehensiveness of those records for patients is improved.

The unimportance of the family and friends and the access to care dimensions of PCC among patients with end-stage renal disease contrasts with previous evidence of their importance in other patient populations as well as the literature on caregivers for patients on dialysis treated at home. Kennedy et al. [28], for example, showed that patients participating in the Patient-Centred Medical Home (PCMH) programme considered efficiency in appointment scheduling and reduction of waiting times to be necessary improvements for PCC. Also patients with intellectual disabilities or mental disorders have been shown to benefit from family interventions; for example, such interventions have improved outcomes for people with early psychosis [29]. These differences may also be related to the nature of care delivery in haemodialysis departments; appointment scheduling could be less important for patients undergoing dialysis because the treatment plan is standardised. In addition, the chronic nature of the disease and the lengthy, intensive (i.e. three times per week) treatment period make that these patients become experts in their own care, potentially reducing their (perceived) dependence on family. However, a growing number of patients with chronic kidney disease receiving home-based care do require support from family and friends to manage their disease [30]. Managing dialysis at home may have a profound and pervasive effect on family and friends and place a heavier toll on their well-being [31], which could make the family and friend dimension more important in this setting.

Respondents’ indifference to the emotional support and the continuity and transition dimensions of PCC may also be typical for this patient population. Patients with an acute, life-threatening disease probably are more likely to be anxious and have strong feelings about emotional support. For example, Mello et al. [32] reported that discussions about emotional support with healthcare professionals increased the odds that cancer survivors would report anxiety and depression. Patients receiving haemodialysis, on the other hand, may feel less anxiety as they are usually dealing with a long-term condition. Noticeable within this dimension is the relatively greater importance attached to patients’ anxiety compared with anxiety in relation to relatives. Finally, the generally low importance of items from the continuity and transition domain of PCC contrasts with Odell [33], who found that a smooth transfer was important to patients moving from the intensive care unit to a general ward. These patients found transfer to be traumatic, confusing, stressful, and tiring; in contrast, patients with end-stage renal disease, who are rarely transferred, attached much lower importance to a smooth transition.

The discussion here above points out that the views on what is important for PCC in a haemodialysis department, as identified in this study, may not be easily transferable to other patient populations, or to other care settings or countries. Previous research has shown that PCC levels and patient outcomes are affected by the setting in which care is delivered, including location, types of illness and treatment, and patient characteristics (e.g. age, gender, ethnicity, and insurance coverage) [4]. Thus, in order to establish the relative importance of PCC dimensions in other care settings, additional research is needed.

This study comes with limitations. Firstly, although the 35 statement representing the 8 dimensions of PCC were carefully developed based on the literature [e.g. 2-9], other researchers might have made different choices or used alternative wordings, leading to a different research instrument. For instance, we did not involve patients and professionals directly in the development of the research instrument, which could have been done using the Delphi technique or conducting focus groups. However, at the end of the interview we did ask respondents whether they felt any important aspect of care was missing from the set of statements; no additional aspects were identified, which provides an indication of the comprehensiveness of the research instrument. Nevertheless, we cannot exclude the possibility that the choices we made in this study may have affected our findings. Still, it is important to bear in mind that in a Q methodology study the focus is on the meaning represented by the relative ranking of the full set of statements, and much less on the (wording of) specific statements (but not trivialising the importance of careful consideration in the development of the statement set). Secondly, we used a non-probability sampling technique (purposive sampling) and a relatively small sample, which fits the method of study. We carefully selected the sampling criteria in order to improve the odds of including respondents representing different views on PCC to participate in this study, and we proceeded with interviewing participants until we felt data saturation was achieved. Still, we cannot exclude that we may have missed people representing alternative views, for instance, related to levels of engagement among patients, level of frailty, comorbidity or time spend on dialysis. Future research will have to explore this.Thirdly, perspectives may change and participants may move from one perspective to another over time. Finally, explored views on what is important for PCC in end-stage renal disease only. As the discussion here above highlighted, investigating views on PCC for other diseases may result in different findings. Future research to investigate views on PCC, if desired using the same set of 35 statements, is needed to increase our understanding of viewpoints in different care settings for different patient populations. This research clearly showed that not all PCC dimensions are equally important. Therefore, healthcare organizations aiming to improve PCC should consider looking into the relative importance of the different dimensions of PCC in their specific context of care provision as this may help improve PCC in the most efficient manner.

## Conclusions

This study identified four views on what is important for PCC among patients and professionals in the haemodialysis department at Maasstad Hospital in Rotterdam, the Netherlands. This finding suggests that no single solution is available for the provision of PCC to patients with end-stage renal disease; different types of care may be needed for different types of patients. Some patients, for example, are expected to benefit from educational interventions to improve their self-management skills and place them in charge of their own care, whereas other patients may benefit more from the availability of a care coordinator to make decisions with them, or for them. Efforts to improve patient outcomes through PCC should therefore be tailored to the heterogeneity in patients’ needs and preferences.

## Abbreviations

PCC: Patient-Centred Care
PCMH: Patient-Centred Medical Home
